# Deep Learning-Based Segmentation to Establish East Asian Normative Volumes Using Multisite Structural MRI

**DOI:** 10.3390/diagnostics11010013

**Published:** 2020-12-23

**Authors:** Regina E. Y. Kim, Minho Lee, Dong Woo Kang, Sheng-Min Wang, Nak-Young Kim, Min Kyoung Lee, Hyun Kook Lim, Donghyeon Kim

**Affiliations:** 1 Research Institute, NEUROPHET Inc., Seoul 06247, Korea; reginaeunyoungkim@neurophet.com (R.E.Y.K.); minho.lee@neurophet.com (M.L.); 2 Institute of Human Genomic Study, College of Medicine, Korea University, Seoul 15355, Korea; 3 Department of Psychiatry, University of Iowa, Iowa City, IA 52240, USA; 4 Department of Psychiatry, Seoul St. Mary’s Hospital, College of Medicine, The Catholic University of Korea, Seoul 06591, Korea; kato7@hanmail.net; 5 Department of Psychiatry, Yeouido St. Mary’s Hospital, College of Medicine, The Catholic University of Korea, Seoul 07345, Korea; smwang11@naver.com (S.-M.W.); nakyoung17@gmail.com (N.-Y.K.); 6 Department of Radiology, Yeouido St. Mary’s Hospital, College of Medicine, The Catholic University of Korea, Seoul 06591, Korea; 22000659@cmcnu.or.kr

**Keywords:** MRI normative, aging, deep-learning, volumetry, multicenter

## Abstract

Normative brain magnetic resonance imaging (MRI) is essential to interpret the state of an individual’s brain health. However, a normative study is often expensive for small research groups. Although several attempts have been made to establish brain MRI norms, the focus has been limited to certain age ranges. This study aimed to establish East Asian normative brain data using multi-site MRI and determine the robustness of these data for clinical research. Normative MRI was gathered covering a wide range of cognitively normal East Asian populations (age: 18–96 years) from two open sources and three research sites. Eight sub-regional volumes were extracted in the left and right hemispheres using an in-house deep learning-based tool. Repeated measure consistency and multicenter reliability were determined using intraclass correlation coefficients and compared to a widely used tool, FreeSurfer. Our results showed highly consistent outcomes with high reliability across sites. Our method outperformed FreeSurfer in repeated measure consistency for most structures and multicenter reliability for all structures. The normative MRI we constructed was able to identify sub-regional differences in mild cognitive impairments and dementia after covariate adjustments. Our investigation suggests it is possible to provide a sound normative reference for neurodegenerative or aging research.

## 1. Introduction

Establishing a normative brain volume is of great importance for clinical assessment and aging studies. A normative reference of neuroimaging data should provide what is usual within a defined population, that is, race and age, at a specific point of the period [1]. Several studies have reported that brain MRI provided a normative reference for researchers and clinicians. Brewer described the utilization of an automated tool to construct normative ranges for volumetric brain MRI using the Alzheimer’s disease neuroimaging initiative (ADNI) dataset [2]. Courchesne et al. investigated the normal brain during development and aging using 116 volunteers aged 19 months to 80 years [3]. Ball et al. focused on the normative MRI for developmental brain, who aged between 4 and 18 years [4]. Potvin et al. provided normative population data for subcortical regional volumes using 2790 healthy individuals aged between 18 to 94 years [5].

While several studies have demonstrated the normative brain as described above, only limited reports are available for East Asian populations. One recent study described Korean normative brain data, but within a limited age range of 65 to 85 years [6]. Another study described mean tissue and lobe volumes in a population with an average age of 59.5 years [7]. Normative data, including younger and wider age ranges, are required for better assessments of normal brain aging. The collection of further data for normative brain MRI, however, is often time-consuming and expensive for individual or small groups of researchers.

In recent years, a growing number of open MRI data are available for researchers to expedite brain research findings. The utilization of these open-source MRI data to create a normative reference will expand the data size and age range with reduced research efforts and costs. Still, these open MRI data are often multicentered and thus could be incompatible with each other due to the heterogeneity of various imaging protocols. Heterogeneity across MRI data is a huge challenge for neuroscientists.

This study investigated whether these open-source multicenter data could establish an East Asian normative reference using an in-house segmentation tool. To test our hypothesis, we gathered two East Asian open-source data that collected MRI from younger healthy participants. These open-source data were collectively analyzed and compared with the data from three study sites, whose participants were in the middle to later ages. This study utilized our in-house deep learning-based automatic segmentation tool, specifically designed for multicenter large-scale MRI segmentation.

Here, we describe the data gathered and then investigate the robustness of extracted subregional volumes for repeated measure consistency and multicenter reliability. We further compared the robustness to the FreeSurfer, a well-established and widely accepted ‘good enough’ tool. Next, we describe the brain subregional distribution across ages 18 to 96 years constructed using a multicenter normative MRI. Lastly, we further describe that the multicenter normative data could identify a statistical difference in brain subregional volumes for mild cognitive impairment (MCI) and dementia, even in age-stratified groups.

## 2. Materials and Methods

### 2.1. Data Description

**Figure 1:**
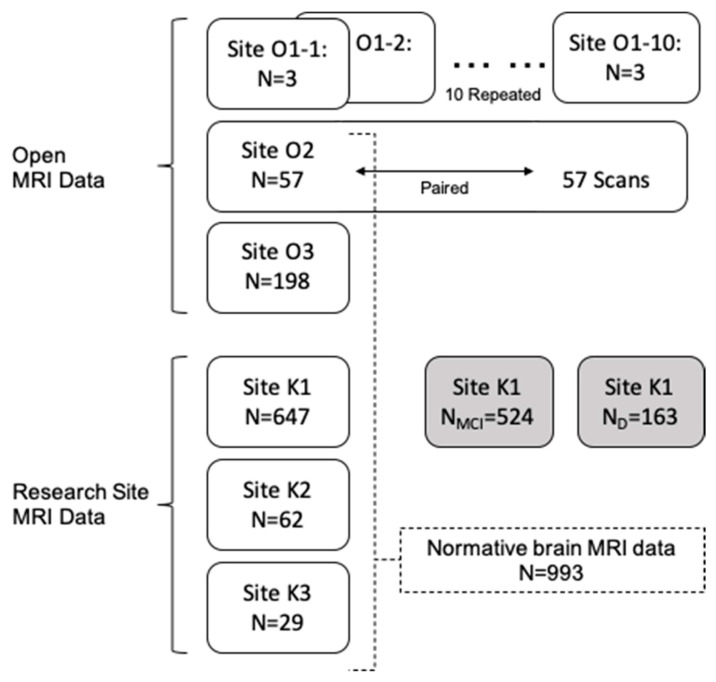
Data sets used in this study are shown by their source and their usage. MRI data from two sources are used: Open MRI data (Site O*N*) and our research site data (Site K*N*). Site O1, consisting of three subjects scanned at 10 (O1-1~O1-10) different sites, was used to investigate multicenter reliability. Site O2 data includes 57 paired scans for the same subjects from the same scanner and used (1) to measure repeated measure consistency and (2) to construct normative data. Sites O2, O3, K1, K2, and K3 are one-time scans from cognitive normal participants. Site K1 additionally includes data for MCI and dementia, compared with our multisite normative data. MCI, mild cognitive impairment; D, dementia.

The dataset used in this study (Figure 1) is largely obtained from three open sources and three research sites. The details of each dataset are described below.

#### 2.1.1. Open MRI Data O1: Multicenter Dataset

A 10-scan set of three subjects was utilized to investigate multicenter measurement reliability. Young healthy participants were scanned in ten scanners traveling participants across sites from October 2016 to November 2017. All the 10 scanners were 3 T MR MAGNETOM Prisma using a 3D magnetization-prepared two rapid acquisition gradient echo (MP2RAGE) sequence, and a detailed study design is published elsewhere [8].

#### 2.1.2. Open MRI Data O2: Repeated Measure Dataset

For the repeated measure consistency assessments, we also used open-source test-retest data for 57 subjects scanned two times at an interval of approximately 6 weeks. All the participants were healthy young adult volunteers aged 19 to 30, recruited from Beijing Normal University. All MRI data were obtained using a SIEMENS Trio Tim 3.0 T scanner and T1-weighted MRI was obtained using a sagittal 3D magnetization prepared rapid gradient echo (MP-RAGE) sequence. The details of the imaging parameters and study design can be found in their descriptive paper [9].

#### 2.1.3. Open MRI Data O3: Chinese Normative Data

From the 1000 Functional Connectomes Project (FCP), 198 MRI scans scanned at the Beijing center were included for the healthy normal modeling. MRI is obtained using an MP-RAGE. A detailed description can be found elsewhere [10].

#### 2.1.4. Research Site Data Sets in Korea

Data from three research sites in Korea were utilized. Data from these three sites are available for 647, 62, and 29 MRI scans from Catholic University of Korea St. Mary’s Hospitals (at Yeouido and Eunpyeong, K1) both using T1 MP-RAGE sequence, Wonkwang University Hospital (K2) using 3D T1 TFE sequence, and Catholic University of Korea Saint Vincent’s Hospital (K3) using 3D T1 MP-RAGE sequence, respectively. In addition, we utilized MCI (*n* = 524) and dementia (*n* = 163) cases from the K1 site. These two cases were investigated in comparison to the normative data gathered from multiple sites.

The study was designed based on the ethical and safety guidelines set forth by the Institutional Review Board of Catholic University of Korea, which approved all research activities. Informed and written consent was obtained from all participants. Our MCI and dementia groups were based on the clinical dementia rating (CDR) scales, where MCI had a CDR of 0.5 and dementia had a CDR of 1 or higher. Although not specified, our dementia condition mostly consists of participants with probable Alzheimer’s disease (AD), who met the National Institute of Neurological and Communicative Disorders and Stroke/Alzheimer’s Disease and Related Disorders Association criteria for probable AD. Participants who had other neuropsychological conditions, such as rarer forms of dementia or traumatic injury, those who were receiving psychotropic medications were excluded. Imaging protocols and other details have been described in a previous study [11].

### 2.2. Brain MRI Segmentation

The entire image processing pipeline was implemented using Python 3.7 with TensorFlow version 1.5.

#### 2.2.1. Preprocessing

We applied an identical pipeline from the pre-processing pipeline for all the data utilized in this study. Our preprocessing includes resampling, zero-padding, and intensity normalization using histogram matching. We first resampled the image in an isotropic voxel (1 mm^3^), then padded it with zero using a filter size of 16 × 16 × 16 and 24 × 24 × 24 for training and testing, respectively. Finally, we normalized the MRI intensity by applying a histogram matching algorithm as described elsewhere [12]. The entire preprocessing pipeline was implemented using the NiftiNet library [13].

#### 2.2.2. Deep Learning Segmentation

**Figure 2:**
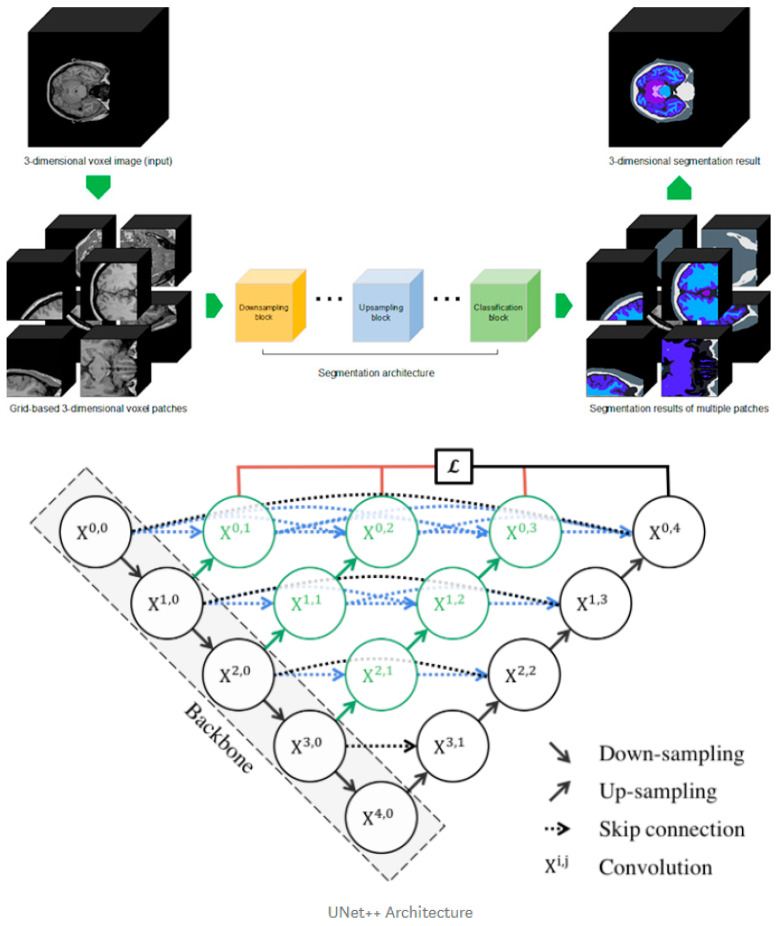
Three-dimensional patch-based training scheme explanation (**upper**) and Improved U-Net++ architecture (**bottom**).

Our in-house segmentation tool was developed from the existing UNet++ deep learning architecture with a three-dimensional methodology to train 104 labels. Our deep learning design has a convolutional layer in the skip path, which bridges the semantic gap between the encoder and decoder characteristic maps. A dense skip connection in the skip path, which improves the gradient flow, has deep supervision, which enables model pruning, improves performance, or, at worst, compares using only one lossy layer (Figure 2, bottom). The cross-entropy loss function was used for the voxel-by-voxel segmentation learning and the learning rate for the Adam optimizer was 0.0001.

For the training dataset, we performed Desikan–Killiany atlas-based FreeSurfer segmentation on 388 patients from public datasets including HCP, ADNI, PPMI, AIBL, and IXI, and two experts performed manual correction to produce a fine-tuned gold standard. Among the entire data set, we first randomly shuffled and set aside 49 datasets for testing. The remaining data were then categorized for training and validation (9.5:0.5). The training data were constructed by extracting the three-dimensional patch image using uniform sampling (96 × 96 × 96) for the individual ground truth data (Figure 2, upper). With the aforementioned training parameters, the model was iteratively trained 500,000 times. The batch size was set to 1, which was the limit that could be handled by the 11 GB RAM of one RTX 2080Ti GPU. For comparison, FreeSurfer software (version 7.0.0, https://surfer.nmr.mgh.harvard.edu) was used to identify subregional brain volumes.

#### 2.2.3. Postprocessing

To increase the validity of the structures, we further applied connected-component labeling [14]. No other postprocessing was applied and volumes with regard to the original MRI were computed for the analysis. The Dice similarity coefficient (DSC) was computed to note the segmentation validity of our method against gold-standard labels edited using FreeSurfer (Table S2).

### 2.3. Statistical Analysis

All analyses were performed using R version 4.0.0, and R package “irr” was used to compute intraclass correlation (ICC). ICC was computed to provide the degree of similarities between repeated volume measurements of the same subjects [15]. Two-way ICC correspondence and agreement were computed for repeated measure consistency and multisite reliability, respectively. Higher ICC values represent better compliance across repeated or multisite measures, and ICC values of 0.75 or higher were suggested to be reliable [15]. Normative data were constructed using data from sites O3 and K1–3. In summary, a total of 993 MRI scans were available for normative data, including ages from 18 to 96. Analysis of variance was conducted to identify statistical differences in demographic characteristics between our constructed normative data, MCI, and dementia. A generalized linear regression model was used to compute the adjusted mean difference in eight regional brain volumes for MCI and dementia when compared to the normative group. From among the entire dataset, age subgroup analyses were conducted to identify age-independent differences between groups in intracranial volumes. The analyses for the age subgroup were only conducted for the 71–80 years and 81–100 years groups due to their limited sample size. Each regression model was adjusted for age at MRI, sex, site, and intracranial volume.

## 3. Results

### 3.1. Repeated Measure Consistency

**Figure 3:**
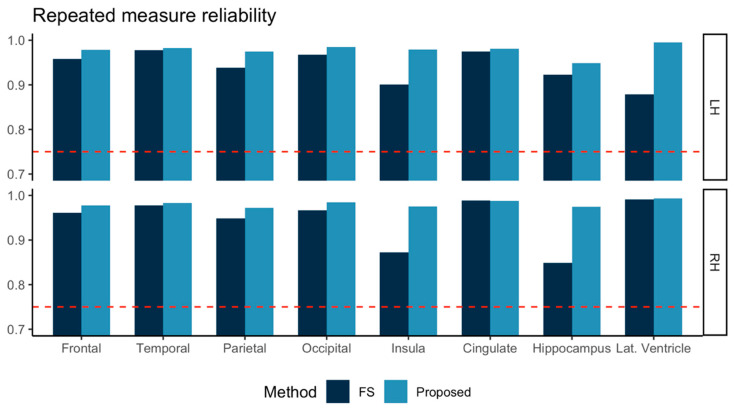
Repeated measure reliabilities for the volumes in eight sub-regions in left and right hemispheres (LH, RH) were evaluated using intraclass correlation (two-way, consistency) for three subjects scanned at 12 different sites. FreeSurfer (FS, dark blue) and the proposed method (light blue) are marked together for comparison. Other than the lateral ventricle (Lat. Ventricle) area, all the volumes were measured for gray matter tissue.

The ICC computed for test-retest MRI of the 57 subjects is shown in Figure 3. Both FreeSurfer and our proposed method achieved ICC > 0.75. In all eight sub-regions investigated, our proposed method showed higher ICC values than those from FreeSurfer.

### 3.2. Multicenter Reliability

**Figure 4:**
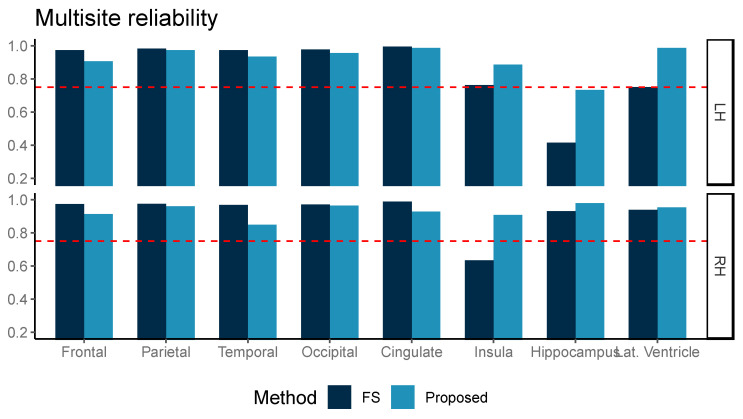
Multicenter reliability, intraclass correlation (ICC, two-way, agreement) was compared between two methods, FreeSurfer (FS) and the proposed method (Proposed, light blue). ICC was computed for two repeated measurements and the reference line at ICC = 0.75 is marked with the dotted red line.

The ICC measured for ten multicenter data of three subjects is shown in Figure 4. Although our proposed method was inferior in ICC values for the frontal, parietal, temporal, and cingulate volumes, the ICC from both methods was above 0.75 for frontal, parietal, temporal, and occipital grey matter volume (GMV) in both hemispheres, the left insula, the right hippocampus, and the right lateral ventricle. Our proposed method further showed ICC > 0.75 in the left lateral ventricle and right insula areas.

### 3.3. Normative Distribution for Adults Ages between 19 and 96 Years

The demographic characteristics of normative modeling are described in the upper left in Table 1. The mean age of the cognitively normal group was 51.4 years, with 64.1% females. The mean years of education was 12.1 years, and the mean intracranial volume (ICV) was 1519.3 mL. The bottom part of Table 1 describes the participants’ characteristics across the five centers.

**Figure 5:**
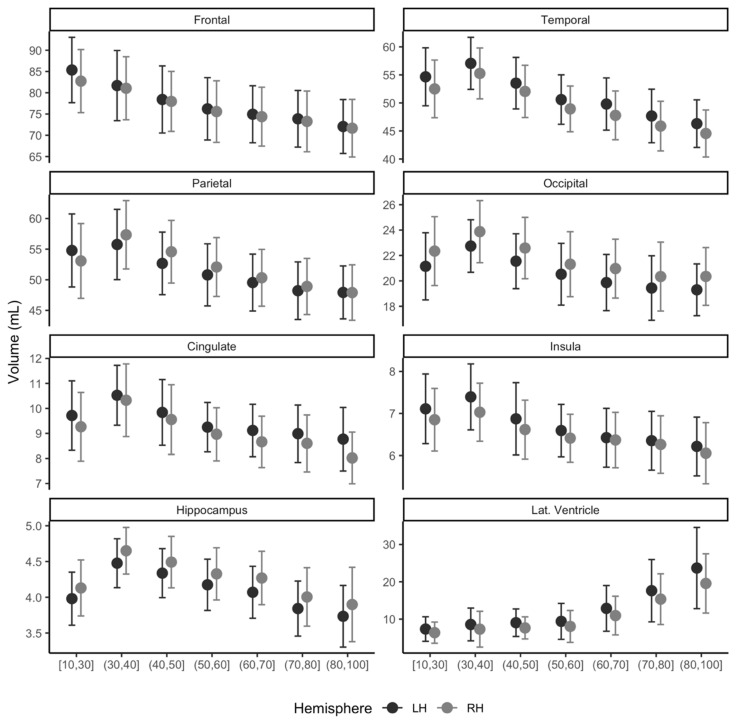
Distribution of regional gray matter (GM) volume (mL) for multicenter normative data of East Asians. Participant ages range from 18 to 96 years and are marked for left (LH, black) and right (RH, gray) hemispheres. Lat. Ventricle, lateral ventricle area volume.

The trend of sub-regional brain volume and age group is shown in Figure 5 for the left and right hemispheres. The frontal lobe and lateral ventricle area showed a consistent decrease or increase in volume with age from 10 s to 90 s in both the left and right hemispheres. Other areas of interest, temporal, parietal, occipital, cingulate, insula, and hippocampus, presented increasing trend in volume until its ages of 30 s and then decreased with age.

### 3.4. Usage of Normative Modeling: Differences of MCI and Dementia in Volumes

Adjusted volume differences of MCI and dementia compared to the constructed multisite normative data are shown in Table 2. Normative data gathered from five centers successfully identified adjusted mean differences in volumes from both MCI and dementia. Significance levels after adjusting for age at MRI, sex, site information, and ICV were mostly *p* < 0.001, except for the left frontal GMV (*p* = 0.002), left occipital GMV (*p* = 0.009), and right occipital (*p* = 0.001) in the MCI group compared to the constructed normative group. Adjusted mean differences in volumes for MCI range from −15 mL for the left insula to +5.68 mL for the left lateral ventricle, where the larger ventricle is often related to age or pathology. For the dementia group, the adjusted volume difference was from −0.36 mL for the left insula and +9.63 mL for the left lateral ventricle. Furthermore, our results also presented a smooth trend in volumes from 10 to 100 years, as shown in Figure 5.

### 3.5. Usage of Normative Modeling: Group Differences in Volumes within Age Subgroups

A similar analysis in age subgroups for 71–80 years and 81–100 years is shown in Table 3. When compared to the cognitive normal controls, the lower volume in the dementia group remained significant for age subgroups of 71–80 and 81–100 years (*p* < 0.05) after adjustments. The difference in MCI, however, slightly differed when an age-subgroup analysis was conducted. The MCI participants in their 71–100 years old showed no difference in the left occipital (71–80 years for *p* = 0.867; *p* = 0.532 for and 81–100 years) and in the right occipital (*p* = 0.238 for 71–80 years and *p* = 0.213 for 81–100 years) lobes. In addition, MCI participants in their 81–100 years further presented no difference in the insula in both hemispheres (*p* = 0.077 and *p* = 0.082 for the left and right areas, respectively) and the left cingulate area (*p* = 0.120) when compared to the same age groups of cognitively normal participants.

## 4. Discussion

This study sought to construct normative brain volumes using MRI collected from multiple centers using our in-house deep learning-based segmentation tool for eight regions of interest (ROIs), frontal, parietal, temporal, occipital, cingulate, insula, hippocampus, and lateral ventricle in the left and right hemispheres. Consistency for repeated measures and reliability for multiple sites were evaluated and compared to those from FreeSurfer. Our proposed segmentation method presented high ICC (>0.75) in general for both repeated measure reliability and multisite reliability (except for the left hippocampus). Our proposed method outperformed FreeSurfer for the insula, hippocampus, and ventricles in multisite reliability and for all eight ROIs in repeated measure consistency. Our multisite-gathered normative data further indicated that the data could be used to identify subregional volumetric differences in MCI and dementia. These large-scale normative data processed through the segmentation tool customized for the multicenter study could help to understand developmental, aging, and pathological changes in the brain.

For the repeated measure reliability investigation, FreeSurfer and our proposed method both achieved ICC values all higher than 0.75. Our proposed method further presented higher ICC than FreeSurfer in all ROIs we investigated. As noted above, a higher ICC means a higher correlation between two independent measurements in a series of data. Repeated MRI used in this study was acquired in a short time interval (<6 weeks), where meaningful biological changes were not expected. Therefore, the measurements obtained from these repeated MRI should indicate no significant differences from each other. Our results indicated that both methods we tested, FreeSurfer and our proposed method, achieved good agreement in terms of ICC (>0.75) and further noted that our proposed method achieved higher agreement than FreeSurfer.

In the multicenter reliability investigation, we found that the ICC was generally similar between our proposed method and FreeSurfer for relatively larger areas, such as the frontal, parietal, temporal, occipital, and cingulate volumes. In addition, our proposed method presented higher ICC than from FreeSurfer in smaller regions of interest, including the insula, hippocampus, and lateral ventricle area in both the left and right hemispheres. This difference in ICC, which is known to be sensitive to intra-method variances as well as inter-method correlation, also reflected improved measurement reliability. Our proposed method achieved high ICC (>0.75), suggesting a cutoff for a good agreement [15], for most ROIs, except for the left hippocampus [15].

Our results indicate that our proposed tool can be used to construct normative data using a multicenter MRI. In this study, we gathered normative data from five different sites with a wide age range from 18 to 96 years. Our mean ICV (1519.3 mL) was compatible with those reported previously (1501.1 mL for older individuals [16]; 1291.0 mL and 1425.0 mL for females and males, respectively [17]). The trend of our normative brain volume across ages is well in line with previous age-associated reports as follows: The decreasing trend of GMV with age has also been well documented in several previous studies [18,19,20]. The hippocampus trajectory across age groups has been reported to be curved across the ages from the 20 s to 100 s [21], and our data showed a similar trend using multicenter gathered normative data (Figure 5).

Our investigation further indicated that the multisite normative data could be used to identify differences in MCI and dementia groups for all ROIs. After adjusting for age at MRI, sex, site information, and ICV, all ROIs were significantly smaller than the reference normative data except for the lateral ventricles. The ventricle areas were significantly larger than the normative group, as the larger ventricle often presents a smaller brain tissue area.

We also conducted age-subgroup analyses to minimize the confounding effects of age, where MCI and dementia tend to have an older age than in cognitively normal participants (e.g., mean ages 51.4 ± 20.9, 75.2 ± 8.2, and 78.9 ± 8.4 years for cognitive normal, MCI, and dementia groups, respectively). The age subgroup analyses of both 71–80 and 81–100 years consistently revealed smaller volumes in the dementia than the cognitive normal for all the ROIs we investigated (*p* < 0.05 after adjustments). In addition, we also showed that the MCI group also had significantly (*p* < 0.05 after adjustments) smaller volumes in most structures, except for the occipital lobe in both age subgroups and the insula and left cingulate area in older age groups of 81–100 years. These results are in line with previous studies reporting that AD-related atrophy is mostly observed in the temporal (mostly medial temporal) area and the hippocampus, followed by the parietal and frontal areas [22]. The difference in the occipital lobe was not significant in our age subgroup analysis, which is also in good agreement with a previous study reporting marginal or no age-related atrophy in the occipital lobe [23]. The older age group also did not show differences in both the insula and left cingulate areas, where all the areas marginally showed dementia-related changes [22,23,24].

The limitations of the present study must be acknowledged before drawing conclusions. First, more segmentation methods have to be incorporated to investigate which is the best method for multisite normative brain MRI studies. Second, further validation datasets may be needed to better understand the capacity and limitations of using multicenter data to construct multisite normative groups. Third, our results may not be applicable to other imaging modalities, such as diffusion-weighted MRI or CT.

## 5. Conclusions

We have presented evidence that multisite brain MRI using a deep learning-based segmentation tool could be used to construct normative brain volume and can identify subregional volumetric differences in MCI and dementia participants. The utilization of multisite data to construct reliable and consistent normative volumes from brain MRI volume could advance brain science and research effectively. A normative MRI dataset could be acquired from multicenter studies and could advance brain science and research effectively.

## Figures and Tables

**Table 1:** Participants description for cognitive normal brain ages from 18 to 96 years.

		Cognitive Normal *n* = 992		MCI *n* = 524	Dementia *n* = 163	*p*
Age, years		51.4 ± 20.9		75.2 ± 8.2	78.9 ± 8.4	<0.001
CDR		0.0 ± 0.0		0.5 ± 0.0	1.3 ± 0.6	<0.001
CDR Sum of box		0.1 ± 0.3		1.8 ± 1.2	7.2 ± 3.5	<0.001
Female, % (*n*)		64.1 (636)		67.7 (354)	70.6 (115)	
Education, years		12.1 ± 4.2		9.5 ± 5.2	8.5 ± 5.6	<0.001
ICV, mL		1519.3 ± 136.5		1487.9 ± 132.5	1476.5 ± 137.3	<0.001
Cognitive normal across centers						
	Site K1		Site K2	Site K3	Site O3	Site O2
	*n* = 647		*n* = 62	*n* = 29	*n* = 198	*n* = 57
Age, years	61.4 (13.8)		62.8 (7.2)	64.0 (8.2)	21.2 (1.8)	23.1 (2.3)
Age, min-max	18–96		51–82	49–78	18–26	19–30
Female, % (*n*)	65.8% (426)		75.8% (47)	34.5% (10)	61.6% (122)	65.8% (30)
Education, years	12.1 (4.4)		10.9 (4.3)	11.4 (4)		14.0 (0.0)
ICV, mL	1501.9 (131.5)		1470.6 (113.9)	1500.7 (130.4)	1571.4 (135.9)	1598.0 (142.2)

Cognitive normal constitutes data from centers K1-3 and O2-3. Mild cognitive impairment (MCI) and dementia participants were all recruited from Site K1. CDR, clinical dementia rating; ICV, intracranial volume; Site K1, Catholic University of Korea Saint Marry Hospitals; Site K2, Wonkwang University hospital; Site K3, Catholic University of Korea Saint Vincent’s hospital; Site O3 1000 Functional Connectomes Project (FCP) at Beijing; Site O2, China 57 for the first visit.

**Table 2:** Adjusted mean atrophy in regional brain volume for mild cognitive impairment (MCI) and dementia when compared to the cognitive normal reference group.

All Age Groups				
	MCI, mL	*p* ^†^	Dementia, mL	*p* ^†^
Frontal L	−0.93 ± 0.30	0.002	−3.21 ± 0.43	<0.001
R	−1.15 ± 0.30	<0.001	−3.25 ± 0.44	<0.001
Temporal L	−2.18 ± 0.21	<0.001	−5.03 ± 0.31	<0.001
R	−2.44 ± 0.22	<0.001	−4.76 ± 0.32	<0.001
Parietal L	−1.23 ± 0.21	<0.001	−2.74 ± 0.31	<0.001
R	−1.60 ± 0.22	<0.001	−3.37 ± 0.32	<0.001
Occipital L	−0.33 ± 0.13	0.009	−1.00 ± 0.18	<0.001
R	−0.42 ± 0.13	0.001	−1.01 ± 0.18	<0.001
Insula L	−0.15 ± 0.03	<0.001	−0.36 ± 0.05	<0.001
R	−0.22 ± 0.03	<0.001	−0.51 ± 0.05	<0.001
Cingulate L	−0.30 ± 0.06	<0.001	−0.68 ± 0.08	<0.001
R	−0.41 ± 0.06	<0.001	−0.74 ± 0.09	<0.001
Hippocampus L	−0.37 ± 0.02	<0.001	−0.66 ± 0.04	<0.001
R	−0.39 ± 0.03	<0.001	−0.65 ± 0.04	<0.001
Lateral ventricle L	5.68 ± 0.52	<0.001	9.63 ± 0.76	<0.001
R	5.12 ± 0.45	<0.001	8.87 ± 0.66	<0.001

^†^ Adjusted mean difference was calculated using a general linear regression model including covariates, age at magnetic resonance imaging (MRI) scan, sex, site information, and intracranial volume (ICV).

**Table 3:** Age categorized analysis.

	Age 71–80			*p* ^†^	Age 81–100			*p* ^†^
	MCI	*p* ^†^	Dementia		MCI	*p* ^†^	Dementia	
Frontal L	−1.52 ± 0.45	**0.001**	−3.84 ± 0.64	**<0.001**	−1.64 ± 0.81	**0.043**	−3.59 ± 0.87	**<0.001**
R	−1.61 ± 0.47	**0.001**	−3.41 ± 0.68	**<0.001**	−1.82 ± 0.79	**0.023**	−3.50 ± 0.86	**<0.001**
Temporal L	−1.51 ± 0.34	**<0.001**	−4.14 ± 0.49	**<0.001**	−2.26 ± 0.64	**0.001**	−4.21 ± 0.69	**<0.001**
R	−1.79 ± 0.36	**<0.001**	−4.30 ± 0.52	**<0.001**	−2.63 ± 0.64	**<0.001**	−3.80 ± 0.69	**<0.001**
Parietal L	−0.61 ± 0.31	**0.047**	−2.16 ± 0.44	**<0.001**	−1.62 ± 0.63	**0.011**	−2.84 ± 0.68	**<0.001**
R	−0.75 ± 0.31	**0.015**	−2.41 ± 0.44	**<0.001**	−1.45 ± 0.54	**0.008**	−2.36 ± 0.59	**<0.001**
Occipital L	−0.03 ± 0.20	0.867	−0.60 ± 0.29	**0.040**	−0.40 ± 0.34	0.238	−0.88 ± 0.36	**0.016**
R	−0.12 ± 0.19	0.532	−0.72 ± 0.28	**0.009**	−0.40 ± 0.32	0.213	−0.83 ± 0.34	**0.017**
Insula L	−0.30 ± 0.09	**0.002**	−0.78 ± 0.14	**<0.001**	−0.28 ± 0.16	0.077	−0.46 ± 0.17	**0.008**
R	−0.44 ± 0.10	**<0.001**	−0.85 ± 0.14	**<0.001**	−0.31 ± 0.18	0.082	−0.48 ± 0.19	**0.015**
Cingulate L	−0.20 ± 0.05	**<0.001**	−0.47 ± 0.08	**<0.001**	−0.16 ± 0.10	0.120	−0.25 ± 0.11	**0.023**
R	−0.22 ± 0.05	**<0.001**	−0.52 ± 0.08	**<0.001**	−0.23 ± 0.10	**0.023**	−0.44 ± 0.11	**<0.001**
Hippocampus L	−0.30 ± 0.04	**<0.001**	−0.57 ± 0.06	**<0.001**	−0.34 ± 0.07	**<0.001**	−0.50 ± 0.08	**<0.001**
R	−0.28 ± 0.05	**<0.001**	−0.53 ± 0.07	**<0.001**	−0.36 ± 0.08	**<0.001**	−0.45 ± 0.08	**<0.001**
Lateral ventricle L	4.84 ± 1.01	**<0.001**	10.45 ± 1.44	**<0.001**	5.00 ± 1.90	**0.009**	6.05 ± 2.04	**0.003**
R	4.14 ± 0.87	**<0.001**	8.39 ± 1.25	**<0.001**	6.37 ± 1.67	**<0.001**	7.51 ± 1.80	**<0.001**

^†^ Adjusted mean difference was calculated using a general linear regression model including covariates, age at magnetic resonance imaging (MRI) scan, sex, site information, and intracranial volume (ICV). *p*-values numbers marked in bold indicate at the significant level of 0.05. MCI, mild cognitive impairment.

## Data Availability

The part of data presented in this study (O1, O2, and O3) are openly available in NITRC (https://www.nitrc.org/frs/shownotes.php?release_id=1902) [8,10] and in Functional Connectomes Project International Neuroimaging Data-Sharing Initiative (https://doi.org/10.15387/fcp_indi.corr.bnu1) [9]. The other part of the data presented in this study (K1, K2, and K3) are available on request from the corresponding author (H.K.L). The data are not publicly available due to the privacy and ethical restrictions.

## Supplementary Materials

The following are available online at https://www.mdpi.com/2075-4418/11/1/13/s1, Table S1: Cognitive normal (CN), mild cognitive impairment (MCI), and dementia by age group and Table S2: Mean and standard deviation of dice similarity coefficients (DSC) between the proposed method and the gold-standard (manually corrected from FreeSurfer).
