# Supplementary material for: Deep Learning-Based Segmentation to Establish East Asian Normative Volumes Using Multisite Structural MRI
Source: Diagnostics (Basel). 2020 Dec 23;11(1):13. doi: 10.3390/diagnostics11010013 (PMC7824436; doi:10.3390/diagnostics11010013)
Supplement: Supplementary file 1 [file diagnostics-11-00013-s001.pdf]

Table S1. Cognitive normal (CN), mild cognitive impairment (MCI), and dementia by age group.

	CN	MCI	Dementia
(40,50], N=66	89% (59)	11% (7)	
Age, y	46.2 ± 2.8	46.6 ± 2.8	
Female, %	51% (30)	58% (4)	
CDR	0.0 ± 0.0	0.5 ± 0.0	
CDRSB	0.2 ± 0.5	1.6 ± 1.0	
Education, y	14.5 ± 3.3	12.0 ± 2.4	
ICV	1524.0 ± 133.3	1471.4 ± 123.8	
(50,60], N=186	83% (154)	13% (25)	4% (7)
Age, y	56.6 ± 2.5	58.1 ± 2.2	57.7 ± 2.4
Female, %	79% (122)	58% (14)	58% (4)
CDR	0.0 ± 0.0	0.5 ± 0.0	1.0 ± 0.0
CDRSB	0.0 ± 0.1	1.0 ± 1.0	6.1 ± 1.6
Education, y	12.5 ± 3.6	12.7 ± 3.0	13.0 ± 4.0
ICV	1468.1 ± 118.0	1510.2 ± 134.5	1556.2 ± 145.8
(60,70], N=385	71% (275)	25% (95)	4% (15)
Age, y	65.4 ± 2.7	66.5 ± 2.5	64.9 ± 3.9
Female, %	68% (188)	58% (58)	58% (15)
CDR	0.0 ± 0.0	0.5 ± 0.0	1.2 ± 0.4
CDRSB	0.0 ± 0.1	1.3 ± 1.0	6.2 ± 3.3
Education, y	11.7 ± 4.3	10.8 ± 4.7	9.5 ± 4.6
ICV	1483.8 ± 117.5	1506.8 ± 142.8	1440.4 ± 166.6
(70,80], N=480	32% (152)	55% (263)	14% (65)
Age, y	74.8 ± 2.7	76.2 ± 2.7	76.6 ± 2.7
Female, %	66% (100)	58% (180)	58% (50)
CDR	0.0 ± 0.0	0.5 ± 0.0	1.3 ± 0.5
CDRSB	0.1 ± 0.2	1.8 ± 1.2	6.8 ± 3.0
Education, y	10.4 ± 5.0	9.4 ± 5.2	7.3 ± 5.5
ICV	1495.5 ± 131.4	1488.1 ± 132.3	1458.2 ± 128.0
(80,100], N=240	13% (30)	56% (134)	32% (76)
Age, y	83.8 ± 3.4	84.2 ± 3.2	85.5 ± 3.8
Female, %	60% (18)	58% (98)	58% (52)
CDR	0.0 ± 0.0	0.5 ± 0.0	1.4 ± 0.6
CDRSB	0.2 ± 0.9	2.2 ± 1.1	7.9 ± 3.9
Education, y	10.9 ± 4.6	8.0 ± 5.3	8.9 ± 5.7
ICV	1510.3 ± 127.5	1470.9 ± 124.8	1492.0 ± 136.0

ICV, Intracranial volume

Table S2. Mean and standard deviation of dice similarity coefficients (DSC) between the proposed method and the gold-standard (manually corrected from FreeSurfer).

DSC	
Frontal L	0.83 $\pm$ 0.13
R	0.81 $\pm$ 0.16
Temporal L	0.79 $\pm$ 0.13
R	0.81 $\pm$ 0.13
Parietal L	0.85 $\pm$ 0.12
R	0.83 $\pm$ 0.14
Occipital L	0.80 $\pm$ 0.12
R	0.79 $\pm$ 0.13
Insula L	0.86 $\pm$ 0.13
R	0.86 $\pm$ 0.13
Hippocampus L	0.87 $\pm$ 0.12
R	0.88 $\pm$ 0.12
Lateral Ventricle L	0.83 $\pm$ 0.18
R	0.83 $\pm$ 0.16

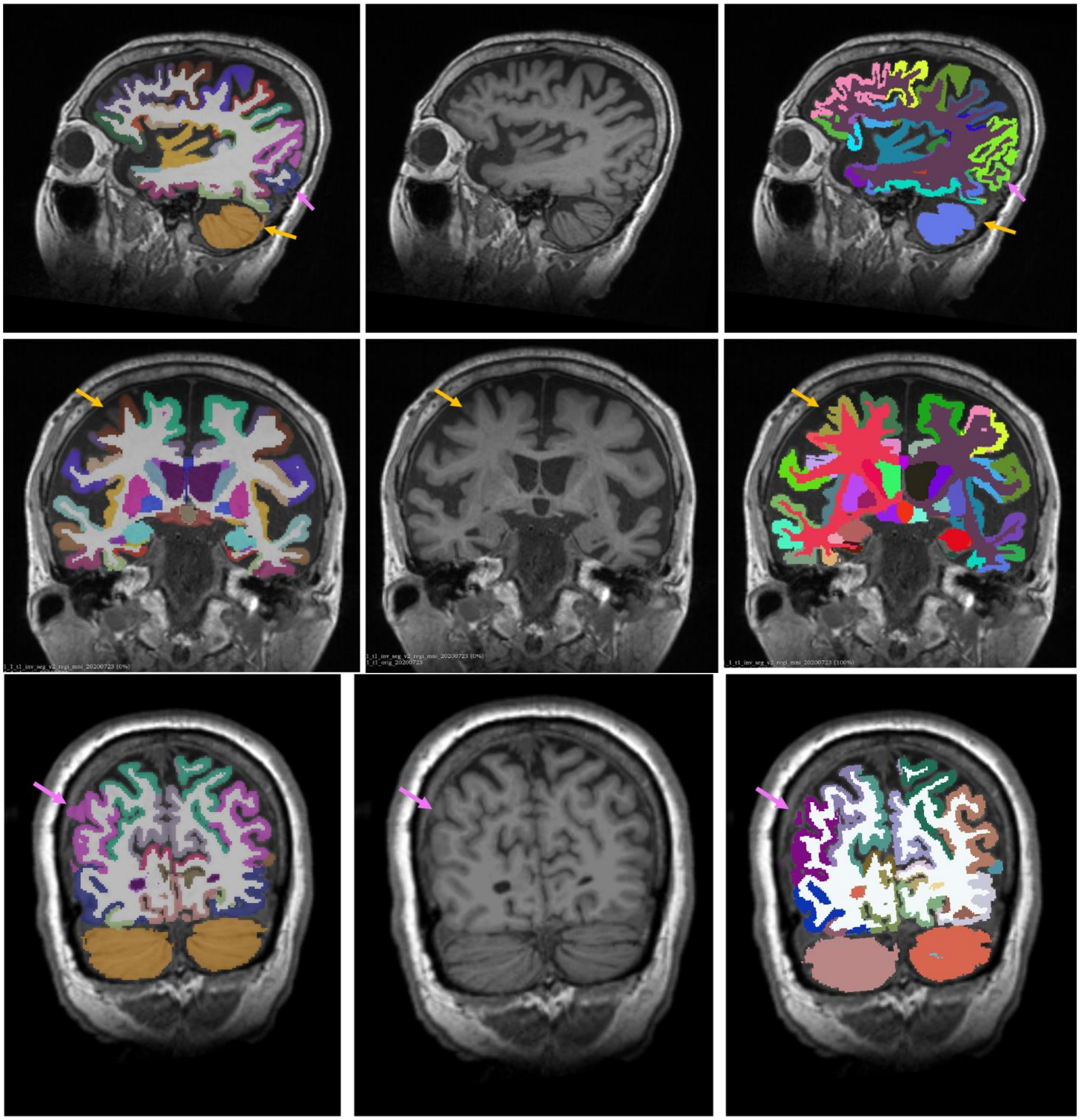

Figure 1. Whole brain segmentation examples with our deep-learning based method (the far left column) and FreeSurfer (the far right column). The T1-weighted image (in the middle) also shown together. The arrow shows slight differences between methods on a same target image.
